# Recurrent SARS-CoV-2 RNA Detection after COVID-19 Illness Onset during Pregnancy

**DOI:** 10.3201/eid2804.212354

**Published:** 2022-04

**Authors:** Isabel Griffin, Kate R. Woodworth, Romeo R. Galang, Veronica K. Burkel, Varsha Neelam, Samantha Siebman, Jerusha Barton, Susan E. Manning, Kathryn Aveni, Nicole D. Longcore, Elizabeth M. Harvey, Van Ngo, Deborah Mbotha, Sarah Chicchelly, Mamie Lush, Valorie Eckert, Paula Dzimira, Ayomide Sokale, Miguel Valencia-Prado, Eduardo Azziz-Baumgartner, Adam MacNeil, Suzanne M. Gilboa, Van T. Tong

**Affiliations:** Centers for Disease Control and Prevention, Atlanta, Georgia, USA (I. Griffin, K.R. Woodworth, R.R. Galang, V.K. Burkel, V. Neelam, E. Azziz-Baumgartner, A. MacNeil, S.M. Gilboa, V.T. Tong);; Minnesota Department of Health, St. Paul, Minnesota, USA (S. Siebman);; Georgia Department of Public Health, Atlanta (J. Barton);; Massachusetts Department of Public Health, Boston, Massachusetts, USA (S.E. Manning);; New Jersey Department of Health, Trenton, New Jersey, USA (K. Aveni);; New York State Department of Health, Albany, New York, USA (N.D. Longcore);; Tennessee Department of Health, Smithville, Tennessee, USA (E.M. Harvey);; Los Angeles County Department of Public Health, Los Angeles, California, USA (V. Ngo); Washington State Department of Health, Tumwater, Washington, USA (D. Mbotha);; Kansas Department of Public Health, Wichita, Kansas, USA (S. Chicchelly);; Nebraska Department of Health and Human Services, Lincoln, Nebraska, USA (M. Lush);; California Department of Public Health, Sacramento, California, USA (V. Eckert);; Pennsylvania Department of Health, Harrisburg, Pennsylvania, USA (P. Dzimira);; Philadelphia Department of Public Health, Philadelphia, Pennsylvania, USA (A. Sokale);; Puerto Rico Department of Health, Bo. Monacillos Río Piedras, Puerto Rico, USA (M. Valencia-Prado)

**Keywords:** COVID-19, coronavirus disease, SARS-CoV-2, severe acute respiratory syndrome coronavirus 2, viruses, respiratory infections, zoonoses, 2019 Novel Coronavirus Disease, real-time reverse transcription PCR, RT-PCR, pregnancy, United States

## Abstract

The Surveillance for Emerging Threats to Mothers and Babies Network conducts longitudinal surveillance of pregnant persons in the United States with laboratory-confirmed severe acute respiratory syndrome coronavirus 2 infection during pregnancy. Of 6,551 infected pregnant persons in this analysis, 142 (2.2%) had positive RNA tests >90 days and up to 416 days after infection.

Detection of severe acute respiratory syndrome coronavirus 2 (SARS-CoV-2) RNA in respiratory specimens that recurs over extended time intervals might indicate viral RNA persistence, continued viral replication, reinfection, or sample testing error (*1*). Although SARS-CoV-2 infections are generally acute, persistent detection of RNA in upper respiratory specimens has been described with a mean duration of 17 days and detectable RNA for up to 12 weeks after symptom onset among recovered patients (*2*–*5*). Virus detection has been reported in severely immunocompromised patients beyond 20 days and up to 143 days after an initial positive SARS-CoV-2 test result (*3*,*4*). SARS-CoV-2 reinfections documented through whole-genome sequencing are rare (*6*).

Immunologic changes during pregnancy might increase risk of SARS-CoV-2 infection, susceptibility to severe illness, and viral shedding (*7*). A cohort study identified several pregnant persons who tested positive 90 days after an initial positive test (*8*). The objective of our study was to describe demographic and clinical characteristics overall and by recurrent test–positive status in a convenience sample of pregnant persons with SARS-CoV-2 infection laboratory confirmed by molecular amplification detection testing (*9*). This exploratory analysis includes data reported from 21 jurisdictions to the Surveillance for Emerging Threats to Mothers and Babies Network; first positive reverse transcription PCR (RT-PCR) results during pregnancy occurred during March 29, 2020–December 31, 2020, with data reported through September 3, 2021.

We enrolled pregnant persons who met our inclusion criteria (Appendix). We defined persons with an RT-PCR–positive respiratory specimen collected >90 days after symptom onset as recurrent positive (RP) independent of the presence of any intermittent RT-PCR–negative specimens. Persons who did not meet the RP definition were labeled not recurrent (NR). However, not all persons received follow-up testing, and additional laboratory results were voluntarily reported. Duration of RT-PCR positivity was defined as the number of days from symptom onset until the last known positive RT-PCR in a respiratory specimen. Duration of RNA shedding was defined as the number of days from symptom onset until the second consecutive negative SARS-CoV-2 result by RT-PCR among pregnant persons reported with a follow-up test. Testing was not routine, and this factor likely will overestimate length of RNA shedding.

Among 6,551 pregnant persons (median age 29 years; 39.7% non-Hispanic White) in our analysis, 17.5% had first trimester infections, 35.5% second, and 47.0% third (Table). Median duration of RT-PCR positivity was 3 days (range 0–416 days). Overall, we collected 9,985 respiratory specimens with RT-PCR results (which could include multiple positive, negative, or indeterminate results per person); 12.5% of specimens tested 90 days after initial symptom onset were positive (158/1,257 specimens) and 9.2% of specimens tested 330 days after initial symptom onset were positive (6/65 specimens). Median duration of documented RNA shedding per person was 130 days (range 0–441 days; n = 458).

**Table:** Symptomatic pregnant persons with SARS-CoV-2 infection detected in respiratory specimens by recurrent positive status, based on RT-PCR positive test result >90 d after symptom onset, Surveillance for Emerging Threats to Mothers and Babies Network, United States, March 29–December 31, 2020*

Characteristic	Total, N = 6,551	Not recurrent, n = 6,409	Recurrent positive, n = 142
Median duration of RT-PCR positivity (range), d	3 (0–416)	3 (0–90)	127 (91–416)
Median duration of RNA shedding (range), d†	135 (0–441)	148 (0–441)	143 (18–412)
Median age at first positive result (IQR), y	29 (25–33)	29 (25–33)	29 (25–32)
Age at initial infection, y‡			
<25	1,547 (23.6)	1,512 (23.6)	35 (25.6)
25–29	2,034 (31.0)	1,990 (31.0)	44 (40.0)
30–34	1,857 (28.4)	1,809 (28.2)	48 (33.8)
>35	1,113 (17.0)	1,098 (17.1)	15 (10.6)
Race/ethnicity			
White non-Hispanic	2,599 (39.7)	2,554 (39.8)	45 (31.7)
Asian non-Hispanic	275 (4.2)	270 (4.2)	5 (3.5)
Black non-Hispanic	1,197 (18.3)	1,171 (18.3)	26 (18.3)
Hispanic	2,077 (31.7)	2,029 (31.7)	48 (33.8)
Multiple or other non-Hispanic	210 (3.2)	200 (3.1)	10 (7.1)
Unknown	193 (2.9)	185 (2.9)	8 (5.6)
Trimester of SARS-CoV-2 infection§			
First	1,146 (17.5)	1,114 (17.4)	32 (22.5)
Second	2,327 (35.5)	2,231 (34.8)	96 (67.6)
Third	3,078 (47.0)	3,064 (47.8)	14 (9.9)
Underlying conditions¶			
Yes	4,945 (74.4)	1,478 (25.6)	10 (7.0)
No	1,488 (22.7)	4,184 (72.4)	131 (92.3)
Unknown	118 (1.8)	117 (2.0)	1 (0.7)

*Values are no. (%) except as indicated. IQR, interquartile range; RT-PCR, reverse transcription PCR; SARS-CoV-2, severe acute respiratory syndrome coronavirus 2. †Duration of RNA shedding was available for only 458 persons. ‡Age in years at initial infection was calculated based on date of first SARS-CoV-2–positive test result during pregnancy or symptom onset if date of positive is unavailable. §Trimester of SARS-CoV-2 infection is calculated based on date of last menstrual period and either date of first positive SARS-CoV-2 results or symptom onset if date of positive unavailable. ¶Presence of any underlying condition was defined as any of the following: obesity, chronic lung disease, hypertension, diabetes, cardiovascular disease, or immunosuppressive conditions (conditions that weaken the immune system [e.g., HIV or AIDS, some cancers such as leukemia] or medications [e.g., chemotherapy or radiation treatment, chronic corticosteroids, or other immunosuppressive medications]).

Overall, 142 persons (2.2%) from 14 jurisdictions met the RP definition. Among 6,409 NR persons, 727 (11.3%) were RT-PCR–negative after 90 days; 5,682 (88.6%) did not have any known RT-PCR results after 90 days. Several RPs were positive by RT-PCR in respiratory specimens for up to 330 days after symptom onset (Figure). Comparing RP to NR persons identified small differences in age group, race/ethnicity, and trimester of infection (Table). A higher percentage of RPs (33.8%) than NRs (28.2%) were 30–34 years of age. Non-Hispanic White persons made up 39.8% of NRs compared with 31.7% of RPs; most RPs were Hispanic (33.8%) and non-Hispanic Black (18.3%). Most RPs had second trimester infections (67.6%), whereas most NRs had third trimester infections (47.8%).

**Figure:**
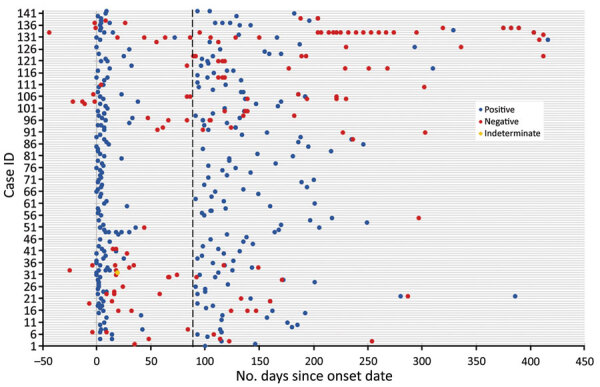
Timing of all known respiratory reverse transcription PCR (RT-PCR) results among recurrent positive pregnant persons (positive test result >90 days from reported symptom onset), Surveillance for Emerging Threats to Mothers and Babies Network, United States, March 29, 2020–December 31, 2020 (n = 142). Data reported as of September 3, 2021. Case ID 4 had positive and negative respiratory RT-PCR tests on day 114; case ID 20 had positive and negative respiratory RT-PCR tests on day 160; case ID 107 had positive and negative respiratory RT-PCR tests on day 186; case ID 135 had positive and negative respiratory RT-PCR tests on days 382 and 389; case ID 139 had positive and negative respiratory RT-PCR tests on day 204. Indeterminate result was defined as one that might indicate the presence of viral RNA but not enough to be considered positive. ID, identification.

Our study’s first limitation is that SARS-CoV-2 genetic sequencing was not reported; reinfection could not be distinguished from recurrent viral shedding. A positive RT-PCR result alone without other information (data on symptom onset, cycle threshold, or viral culture) cannot distinguish infectious virus from noninfectious genomic fragments. Second, health departments were not required to send laboratory testing beyond the first positive result that occurred during pregnancy; these findings are from a convenience sample of pregnant persons with varied duration of follow-up and do not estimate the actual extent of recurrent positivity. Furthermore, persons with infections earlier in pregnancy might be more likely to be classified as RP, given that they can be followed longer and receive additional COVID-19 testing compared with persons with initial symptom onset later in pregnancy. Last, only 31.4% of our cohort (n = 2,062) had multiple test results, representing a small proportion of pregnant persons.

The findings of this report suggest that specimens from pregnant persons diagnosed with symptomatic SARS-CoV-2 infections might be recurrently positive for up to 416 days after symptom onset. Future prospective cohort studies among pregnant persons with SARS-CoV-2 testing should be performed over consistent lengths of time, distinguish infectious viral shedding from noninfectious recurrent positive PCR results, and examine risk of reinfection during pregnancy given the recent emergence of new coronavirus disease variants. Longitudinal surveillance of pregnant persons with COVID-19 can be used for hypothesis generation, in addition to monitoring the impact of infection on pregnancy and infant outcomes.

Appendix Additional information about recurrent SARS-CoV-2 RNA detection after COVID-19 illness onset during pregnancy.
