# Supplementary material for: Recurrent SARS-CoV-2 RNA Detection after COVID-19 Illness Onset during Pregnancy
Source: Emerg Infect Dis. 2022 Apr;28(4):873–6. doi: 10.3201/eid2804.212354 (PMC8962892; doi:10.3201/eid2804.212354)
Supplement: Appendix — Additional information about recurrent SARS-CoV-2 RNA detection after COVID-19 illness onset during pregnancy. [file 21-2354-Techapp-s1.pdf]

Article DOI: https://doi.org/10.3201/eid2804.212354

Recurrent SARS-CoV-2 RNA Detection after COVID-19 Illness Onset During Pregnancy

Appendix

Supplemental Methods

Study inclusion criteria were 1) first reverse transcription PCR (RT-PCR)–positive respiratory specimen taken during pregnancy, 2) age of 10–53 years, 3) known coronavirus disease symptom onset date, 4) known trimester of infection, and 5) known date of first positive respiratory specimen. This activity was reviewed by the Centers for Disease Control and Prevention, determined to be a nonresearch, public health surveillance activity, and was conducted consistent with applicable federal law and Centers for Disease Control and Prevention policy (*1*).

Supplemental Results

Seven percent (7.0%) of recurrent positive (RP) persons had >1 underlying condition (obesity, chronic lung disease, hypertension, diabetes, cardiovascular disease, or immunosuppressive condition) compared with 25.6% of not recurrent persons (Table in main text, https://wwwnc.cdc.gov/EID/article/28/4/21-2354-T1.htm). We hypothesized that RP persons would have other underlying conditions that would affect their immune status besides pregnancy, but this finding could also be explained by the fact that the not recurrent cohort likely contained some undetected RPs. In all but 1 of the 142 RP persons, symptom onset occurred before known circulation (December 20, 2020) of severe acute respiratory syndrome coronavirus 2 variants of interest or concern (*2*).

References

1. Department of Health and Human Services. 45 C.F.R. part 46, 21 C.F.R. part 56; 42 U.S.C. Sect. 241(d); 5 U.S.C. Sect. 552a; 44 U.S.C. Sect. 3501 et seq [cited 2021 Feb 4]. https://www.hhs.gov/ohrp/sites/default/files/ohrp/policy/ohrpregulations.pdf

2. Paul P, France AM, Aoki Y, Batra D, Biggerstaff M, Dugan V, et al. Genomic surveillance for SARS-CoV-2 variants circulating in the United States, December 2020–May 2021. MMWR Morb Mortal Wkly Rep. 2021;70:846–50. PubMed https://doi.org/10.15585/mmwr.mm7023a3
